# Comparison of abdominal and minimally invasive radical hysterectomy in patients with early stage cervical cancer

**DOI:** 10.7150/ijms.55017

**Published:** 2021-01-19

**Authors:** Sang Il Kim, Jiwoo Lee, Jiyun Hong, Sung Jong Lee, Dong Choon Park, Joo Hee Yoon

**Affiliations:** 1 Department of Obstetrics and Gynecology, St. Vincent's hospital, College of Medicine, The Catholic University of Korea, Seoul, Republic of Korea; 2 Department of Obstetrics and Gynecology, Seoul St. Mary's hospital, College of Medicine, The Catholic University of Korea, Seoul, Republic of Korea

**Keywords:** cervical cancer, minimally invasive surgery, laparoscopic surgery, radical hysterectomy, LACC trial

## Abstract

Purpose: The aim of this study was to compare survival outcomes of open radical hysterectomy and minimally invasive radical hysterectomy (MIS) in early stage cervical cancer.

Methods: A retrospective analysis of 148 patients with stage IB1 - IIA2 cervical cancer who underwent either minimally invasive or open radical hysterectomy. Tumor characteristics, recurrence rate, disease-free survival (DFS), and overall survival (OS) were compared according to surgical approach.

Results: In total, 110 and 38 patients were assigned to open surgery and MIS groups. After a medical follow-up of 42.1 months, the groups showed similar survival outcomes (recurrence rate, DFS, and OS). However, in patients with tumor size >2 cm, recurrence rate was significantly higher in MIS group (22.5% vs 0%; p=0.008). And in patients with tumor size >2 cm, MIS group showed significantly poorer DFS than open surgery group (p=0.017), although OS was similar between the two groups (p=0.252).

Conclusion: In patients with tumor size >2 cm, MIS was associated with higher recurrence rates and poorer DFS than open surgery. However, in patients with tumor size ≤2 cm, MIS did not seem to compromise oncologic outcomes.

## Introduction

Cervical cancer is the most common gynecologic cancer in developing countries, accounting for 444,500 new cases and 230,200 deaths each year 1. Approximately 13,800 new cases and 4,290 deaths related to cervical cancer are expected to occur in the United States in 2020 2. In Korea, the incidence of cervical cancer has been decreasing; however, it is still the most common gynecologic cancer. It is expected to account for 3,148 new cases and 801 deaths in 2020 3,4.

In 2018, the prior 2014 International Federation of Gynecology and Obstetrics (FIGO) staging system for cervical cancer was revised. Stage IB disease is now divided into 3 substages and lymph node status is incorporated into stage IIIC disease 5.

Radical hysterectomy with pelvic lymph node dissection is the standard treatment for stage IA1 with lymphovascular space involvement (LVSI) to stage IB2 and IIA1 cervical cancer, and radical hysterectomy with adjuvant therapy is a treatment option for stage IB3 and IIA2 cervical cancer with a bulky tumor 6. Laparoscopic radical hysterectomy was first described in 1992 by Nezhat et al. and Canis et al. 7,8. After minimally invasive surgery (MIS) was introduced for cervical cancer, its incidence compared to open surgery has gradually increased 9. Many studies have shown similar oncologic outcomes for MIS and open surgery 10, 11. However, in November 2018, results from the Laparoscopic Approach to Cervical Cancer (LACC) trial indicated that patients undergoing minimally invasive radical hysterectomy have a lower rate of progression-free survival and overall survival than those who undergo abdominal radical hysterectomy 12. Despite the controversies surrounding the LACC trial, its results are extremely important, and can cause a paradigm shift in how cervical cancer is managed. Based on the LAAC trial, the National Comprehensive Cancer Network (NCCN) and the European Society of Gynaecological Oncology (ESGO) changed the treatment guidelines for early stage cervical cancer 6, 13. Thus, we decided to evaluate the data from our institution with the objective of comparing the risks of recurrence and survival in a cohort of women undergoing minimally invasive radical hysterectomy versus abdominal radical hysterectomy for early stage cervical cancer in a single institution.

## Material and Methods

This retrospective cohort study was performed with approval from the institutional review board of The Catholic University of Korea (No.VC20RISI0080). Informed consent was waived for this study considering its retrospective nature.

### Study population

From our institution's cancer registry, we identified patients who underwent radical hysterectomy for cervical cancer from January 2010 to December 2019 at St. Vincent Hospital. Using FIGO staging system, 274 patients that received primary surgical treatment for pathologically confirmed stage IB1-IIA2 disease were initially included.

We excluded patients with any of the following characteristics from our analysis: (1) underwent incomplete surgical staging; (2) underwent fertility-sparing surgery, or vaginal total hysterectomy; (3) received radiation therapy or neoadjuvant chemotherapy prior to surgery; (4) had histologic types other than squamous cell carcinoma, adenocarcinoma, or adenosquamous carcinoma; and (5) had insufficient clinical and/or pathologic data. Following the revised 2018 FIGO staging, we excluded patients with pathologically confirmed parametrial invasion, which is stage IIB, and pelvic and/or para-aortic lymph node metastasis, which is stage IIIC.

We divided patients that met the study inclusion and exclusion criteria into two groups: those who underwent abdominal radical hysterectomy (open surgery group) and those who underwent minimally invasive radical hysterectomy (MIS group). Robot-assisted surgery was included in the MIS group.

In our patients, after surgery, adjuvant therapy was selectively implemented according to the stage and presence of risk factors such as involvement of resection margin, LVSI, stromal invasion, and tumor size. The uterine manipulator was used during the surgery on case by case basis. The diagnosis of cervical cancer was confirmed histologically in all patients before surgery. Patients diagnosed with cervical cancer commonly underwent preoperative magnetic resonance imaging (MRI) and/or positron emission tomography-computed tomography (PET-CT) and/or computed tomography (CT) to assess the local tumor extent, lymph node metastasis, and distant spread.

### Data collection and definitions

The following data were reviewed: medical records, pathologic reports, and imaging studies (MRI, CT, and PET-CT). We collected information about clinicopathologic characteristics (age, histologic type, grade, FIGO stage, tumor size, and risk factors) and adjuvant treatments such as radiation, concurrent chemoradiation, or chemotherapy. The tumor size was documented as the longest diameter based on either the histopathological findings or preoperative imaging. Overall survival (OS) was defined as the duration in months from the date of initial diagnosis to the date of cancer-related death or last follow-up. Disease-free survival (DFS) was defined as the duration in months from the date of surgery to the date of recurrence based on imaging findings or tissue biopsy.

### Statistical analysis

Differences in clinicopathologic characteristics were evaluated between the two groups. We used Student's t-test, chi-square test, or Fisher's exact test to compare variables. We used Kaplan-Meier methods with log-rank test to compare DFS and OS between the two groups. All statistical analyses were performed using SPSS statistical software (version 21.0; SPSS Inc., Chicago, IL, USA). Statistical significance was set at p<0.05.

## Results

In total, 148 patients met the inclusion and exclusion criteria. Of these, 38 patients underwent open surgery (25.7%), and 110 underwent MIS (74.3%). In the MIS group, 90 patients underwent traditional laparoscopy (81.8%) and 20 underwent robotic surgery (18.2%).

The clinicopathologic characteristics of the patients are presented in Table 1.

The mean age was 49 years in the open surgery group and 52 years in the MIS group. There was no significant difference in the age of patients between the two groups, with older patients being more likely to undergo MIS (p=0.178). Both groups did not show a significant difference in the histologic subtype (p=0.777). Most patients in both groups were in FIGO stage IB1 and IB2 (MIS group, 86.3%; open group, 76.3%; p=0.147). The rate of patients in stage IB1 was significantly higher in the MIS group than in the open surgery group (62.7% vs. 26.3%, p<0.0001), and the rate of patients in stage IB2 was significantly higher in the open surgery group than in the MIS group ((50.0% vs. 23.6%, p=0.002). The rate of loop electrosurgical excision procedure (LEEP) conization before surgery was significantly higher in the MIS group (p=0.0013). LVSI was not significantly different between the two groups (p=0.833). Tumor size was significantly larger in the open surgery group (p<0.0001), and deep stromal invasion was significantly more frequent in the open surgery group (p=0.0009). The open surgery group had a higher rate of patients receiving adjuvant therapy than the MIS group (68.4% vs. 40.0%, p = 0.0025).

**Figure 1:**
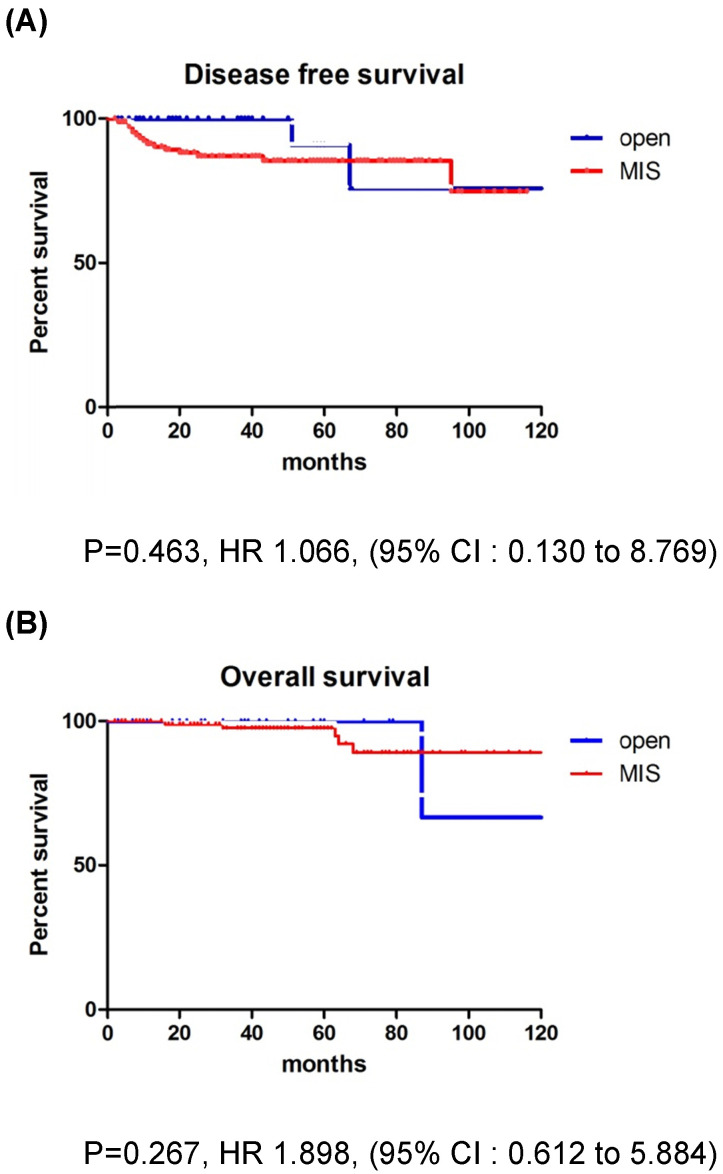
Survival outcomes in study population. All patients. **(A)** disease-free survival, **(B)** overall survival

The mean follow-up time showed no significant difference between the groups (MIS, 45.5 months; open surgery, 37.8 months; p=0.179). There were 17 recurrences (11.5%) in the entire cohort at the time of analysis (Table 2). Recurrences occurred in 15 (13.6%) of the 110 MIS patients and 2 (5.3%) of the 38 open surgery patients. The recurrence rate was higher in the MIS group, but there was no significant difference between the two groups (p=0.179). In the open surgery group, all recurrences occurred in the retroperitoneal nodes. In the MIS group, most of the recurrences were locoregional. There were 6 (4.1%) cancer-related deaths in the entire cohort, 5 (4.5%) in the MIS group, and 1 (2.6%) in the open surgery group (p=0.606). DFS (36.7 vs 45.5 months, p = 0.267), and OS (37.8 vs 50.4 months, p = 0.952) were similar between the MIS and open surgery groups (Fig. 1).

No differences were found between conventional laparoscopy and robotic surgery (Table 3).

**Figure 2:**
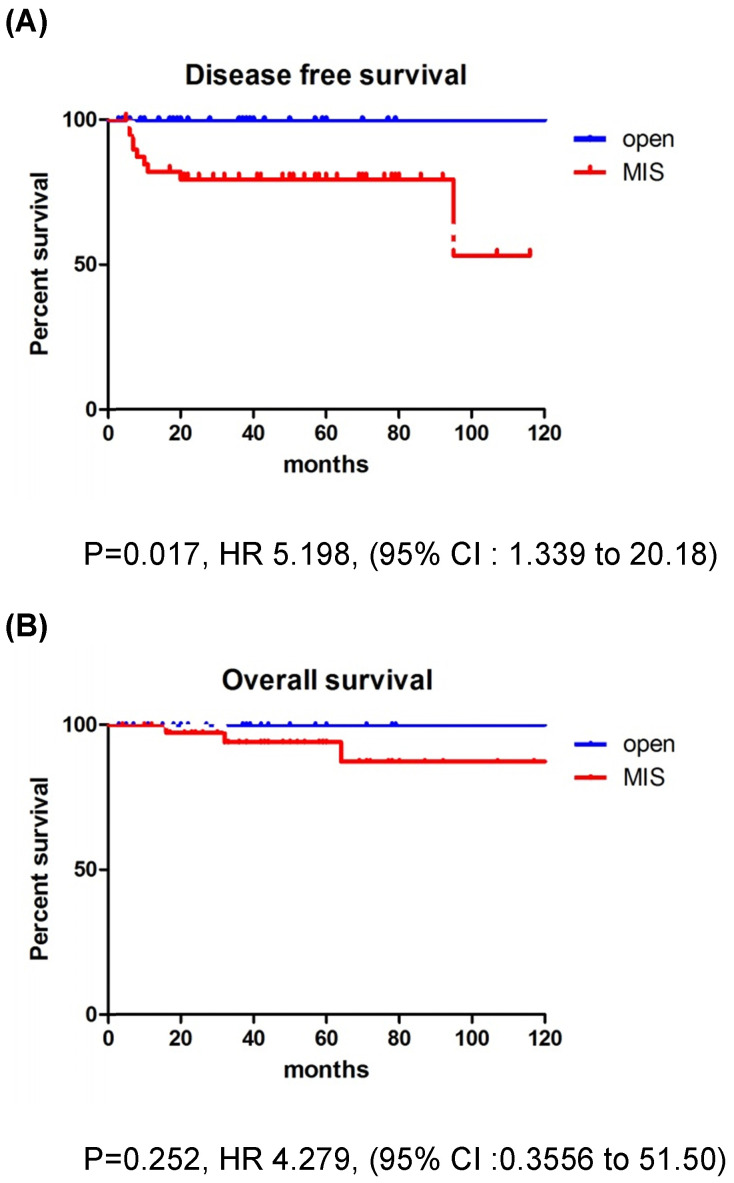
Survival outcomes in study population. Patients with tumor size >2 cm. **(A)** disease-free survival, **(B)** overall survival

The recurrence rate was significantly higher in the MIS group for patients with tumors >2 cm. In addition, patients with deep stromal invasion showed significantly higher recurrence rates in the MIS group compared to the open surgery group. LEEP conization before surgery did not affect survival outcomes. There were no differences between the MIS and open surgery groups, regardless of the adjuvant therapy use. None of the patients with recurrence in the open surgery group received adjuvant treatment, while 6 of the 15 patients with recurrence in the MIS group received adjuvant treatment. Compared to CCRT group, RT only group showed higher recurrence rate in MIS group (21.4% vs. 10.0%). But, there was no significant difference between the two groups (p=0.068) (Table 4). In patients with tumors >2 cm, the MIS group had significantly poorer DFS than the open surgery group (p=0.017; Fig. 2), although OS was similar between the two groups (p=0.252; Fig. 2).

## Discussion

The use of MIS in gynecologic oncology has gradually increased since the first report in 1992 14. Compared to open surgery, MIS is superior in terms of reduced operative morbidity and postoperative complications 15. Additionally, in numerous retrospective studies, MIS showed comparable survival outcomes 10, 11, 16, 17. However, these results were completely contradicted by the LACC trial 12. Although there are some controversies surrounding the results of the LACC trial, this is the only randomized clinical trial comparing survival outcomes between MIS and open surgery 18, 19. The unexpected results from the LACC trial led the NCCN and ESGO to change the treatment guidelines for early stage cervical cancer, which influenced the treatment protocol at many centers 6, 13.

In this hospital-based retrospective analysis, we compared the oncologic outcomes of MIS and open surgery for the treatment of early stage cervical cancer. In our overall cohort, we observed some differences in clinicopathologic characteristics between the two groups. The majority of patients in MIS group were in stage IB1, whereas they were in stage IB2 in the open surgery group. Among the intermediate risk factors, tumor size and the rate of deep stromal invasion were significantly different between the two groups. This led to a significant difference in the rate of adjuvant treatment.

In our overall cohort, which included tumors of all size, both DFS and OS showed comparable outcomes between the groups. These findings contradict the results of the LACC trial. However, in patients with tumor size >2 cm, our results were consistent with the results of the LACC trial; the MIS group showed a significantly higher recurrence rate and worse DFS than the open surgery group.

In our overall cohort, the recurrence rates were 5.3% in the open surgery group and 13.6% in the MIS group. Recurrence rates were higher than those in the LACC trial in both groups. In the LACC trial, recurrence rates were 2.2% and 8.5%, respectively 12. The different results can be explained as we had included patients in stage IIA2 and IB3 with bulky tumor mass**.** In patients with tumor size >2 cm, 9 of 40 patients (22.5%) in the MIS group experienced recurrence. Conversely, no recurrence was found in the open surgery group. Stump recurrences were more frequent among recurrences in the MIS group in the overall cohort. Further, in patients with tumor size > 2 cm, 8 of the 9 recurrences occurred in the vaginal stump. Higher recurrence rates in the MIS group and high rates of stump recurrence can be explained by several factors. The first factor is the use of uterine manipulator. This might cause a breakdown of the tumor and promote dissemination. Second, intracorporeal colpotomy can cause tumor exposure and promote dissemination. In our study, a uterine manipulator was used on case by case basis, and intracorporeal colpotomy was used in most cases. These surgery- or surgeon-related factors may cause an increased risk of recurrence in the MIS group*. Kanao et al.* reported a no-look no-touch technique to prevent intraoperative tumor spillage. The no-look no-touch technique includes the following measures: First, creation of a vaginal cuff; second, manipulation of the uterus without insertion of a uterine manipulator; third, minimal handling of the uterine cervix; and last, bagging the specimen. They compared the surgical and oncologic outcomes of total laparoscopic radical hysterectomy with the no-look no-touch technique to abdominal radical hysterectomy; surgical outcomes were superior in the former technique, and oncologic outcomes were similar in both techniques 20.

Our results indicate that MIS had significantly poorer DFS in patients with tumor size >2 cm. These results are in concordance with a previous study by *Kim et al.* that analyzed survival outcomes among patients with stage IB1-IIA2; patients with MIS had significantly poorer DFS in the subgroup with tumor size >2 cm 21. Our results suggest that before making a decision on the route for radical hysterectomy, tumor size must be considered, and MIS must be implemented in patients with a tumor size ≤2 cm.

Our study has several limitations. First, due to the retrospective study design, there may have been inevitable issues such as selection bias. Second, the sample size may have been insufficient to properly compare DFS and OS between the two groups. Third, due to the surgeon's affinity for MIS, the rate of MIS was extremely high. Fourth, variations in technique, expertise, and outcomes among surgeons were not considered. Fifth, operative morbidity according to the surgical approach was not evaluated.

In conclusion, in patients with tumor size >2 cm, MIS was associated with higher recurrence rates and poorer DFS than open surgery. Thus, this retrospective study supports the results of the LACC trial. However, in patients with a tumor size ≤2 cm, MIS did not seem to compromise oncologic outcomes. Thus, MIS should be performed in highly selected patients with careful implementation of the surgical technique. Additionally, before making a decision on the route for radical hysterectomy, the results of the LACC trial should be informed to every patient.

## Author Contributions

Conception & design of study: SI Kim, SJ Lee, JH Yoon; Data collection: SI Kim, J Lee, J Hong; Responsible surgeon: DC Park, SJ Lee, JH Yoon; Data analysis: SI Kim, J Lee; Manuscript writing/editing: SI Kim, J Lee; Supervision: DC Park, SJ Lee, JH Yoon.

## Figures and Tables

**Table 1:** Clinopathological characteristics of patients according to surgical approach

	Total (n = 148, %)	Open (n = 38, %)	MIS (n = 110, %)	P value
**Age, years**				
Mean ± SD	50.9 ± 10.7	48.9 ± 10.1	51.6 ± 10.8	0.178
**Histologic type**				0.777
SCC	104 (70.3)	27 (71.1)	77 (70.0)	
ACC	38 (25.7)	10 (26.3)	28 (25.5)	
ASC	6 (4.0)	1 (2.6)	5 (4.5)	
**FIGO stage**				0.147
IB1	79 (53.4)	10 (26.3)	69 (62.7)	<0.0001
IB2	45 (30.4)	19 (50.0)	26 (23.6)	0.002
IB3	16 (10.8)	6 (15.8)	10 (9.1)	0.362
IIA1	6 (4.1)	2 (5.3)	4 (3.6)	0.647
IIA2	2 (1.3)	1 (2.6)	1 (1.0)	0.449
**LEEP conization**	41 (27.7)	3 (7.9)	38 (34.5)	0.0013
**Tumor size (cm)**	2.44 ± 1.37	3.19 ± 1.32	2.15 ± 1.27	< 0.0001
**LVSI (+)**	25 (16.9)	6 (15.8)	19 (17.3)	0.833
**Deep stromal invasion**	67 (45.3)	26 (68.4)	41 (37.3)	0.0009
**Adjuvant treatment**				0.0025
None	78 (52.7)	12 (31.6)	66 (60.0)	
Yes	70 (47.3)	26 (68.4)	44 (40.0)	
**Type of adjuvant treatment**				0.853
CCRT	47 (31.8)	17 (44.7)	30 (27.3)	
Radiotherapy	23 (15.5)	9 (23.7)	14 (12.7)	
**Mean follow-up time (months)**	42.1	37.8	45.5	0.179

SD, standard deviation; SCC, squamous cell carcinoma; ACC, adenocarcinoma; ASC, adenosquamous carcinoma; FIGO, International Federation of Gynecology and Obstetrics; LEEP, loop electrosurgical excision procedure; LVSI, lymphovascular space invasion; CCRT, concurrent chemoradiation therapy

**Table 2:** Oncologic survival outcomes according to surgical approach

	Total (n = 148, %)	Open (n = 38, %)	MIS (n = 110, %)	P value
**Recurrence**				
Total	17 (11.5)	2 (5.3)	15 (13.6)	0.163
IB1	8/79 (10.1)	2/10 (20.0)	6/69 (8.7)	0.266
IB2	4/45 (8.9)	0/19	4/26 (15.4)	0.126
IB3	4/16 (25.0)	0/6	4/10 (40.0)	0.496
IIA1	0/6	0/2	0/4	-
IIA2	1/2 (50.0)	0/1	1/1 (100)	-
**Site of recurrence, total**	17	2	15	0.261
Stump	9 (52.9)	0	9 (60.0)	
Pelvic lymph node	5 (29.4)	2 (100)	3 (20.0)	
Lung	2 (11.8)	0	2 (13.3)	
Peritoneum	1 (5.9)	0	1 (6.7)	
**Death**	6 (4.1)	1 (2.6)	5 (4.5)	0.606

**Table 3:** Robotic versus conventional laparoscopy

	Conventional (n = 90, %)	Robotic (n = 20, %)	P value
**FIGO stage**			0.076
IB1	52 (57.8)	17 (85.0)	
IB2	25 (27.8)	1 (5.0)	
IB3	8 (8.9)	2 (10.0)	
IIA1	4 (4.4)	0	
IIA2	1 (1.1)	0	
**Recurrences, total**	14 (15.6)	1 (5.0)	0.297
**Recurrences by FIGO stage**			
IB1	6/52 (11.5)	0/17	-
IB2	4/25 (16.0)	0/1	-
IB3	3/8 (37.5)	1/2 (50.0)	0.747
IIA1	0/4	0	-
IIA2	1/1 (100)	0	-
**Median follow-up (months)**	55.0	43.0	0.059
Range	2-126	2-86	
**Death**	4 (4.4)	1 (5.0)	0.914

FIGO, International Federation of Gynecology and Obstetrics

**Table 4:** Factors associated with oncologic survival outcomes according to surgical approach

	Total (n = 148) Recurrence rate	Open (n = 38) Recurrence rate	MIS (n = 110) Recurrence rate	P value
**LEEP conization**				
No	12/107 (11.2)	2/35 (5.7)	11/72 (15.3)	0.214
Yes	5/41 (12.2)	1/3 (33.3)	4/38 (10.5)	0.330
**Tumor size (cm)**				
≤2 cm	8/80 (10.0)	2/10 (20.0)	6/70 (8.6)	0.259
>2 cm	9/68 (13.2)	0/28	9/40 (22.5)	0.008
**LVSI**				
Negative	15/123 (11.9)	2/32 (6.7)	13/91 (14.3)	0.232
Postitive	2/25 (8.0)	0/6	2/19 (10.5)	0.407
**Deep stromal invasion**				
No	9/81 (11.1)	2/12 (16.7)	7/69 (10.1)	0.507
Yes	8/67 (11.9)	0/26	8/41 (19.5)	0.045
**Adjuvant treatment**				
No	11/78 (14.1)	2/12 (16.7)	9/66 (13.6)	0.674
Yes	6/70 (8.6)	0/26	6/44 (13.6)	0.078
**Type of adjuvant treatment**				
CCRT	3/47 (6.4)	0/17	3/30 (10.0)	0.292
Radiotherapy	3/23 (13.0)	0/9	3/14 (21.4)	0.253

LEEP, loop electrosurgical excision procedure; LVSI, lymphovascular space invasion; CCRT, concurrent chemoradiation therapy
